# A Case of *Sparganosis mansoni* in the Thigh: Serological Validation of Cure Following Surgery

**Published:** 2012

**Authors:** T Chiba, Y Yasukochi, Y Moroi, M Furue

**Affiliations:** 1 Dept. of Dermatology, Iizuka Hospital, Fukuoka, Japan; 2 Dept. of Dermatology, Graduate School of Medical Sciences, Kyushu University, Japan

**Keywords:** Sparganosis, Surgery, ELISA, Ultrasonography

## Abstract

Cases of *Sparganum mansoni*, caused by the plerocercoid larva of the tapeworm *S. mansoni*, occur throughout the world, particularly in Asian, Middle Eastern, and European countries. However, cases of infection with this parasite are rarely seen in Japan. Here, we present a case of a 61-year-old woman with a solitary subcutaneous nodule in left inner aspect of the thigh, from which a long, slender, whitish worm was surgically removed. The parasite was histopathologically identified as *S. mansoni*. Serological testing confirmed cure of the infection after surgical removal of the parasite. The authors advocate immunoserological examination in case of *S. mansoni*.

## Introduction

*Sparganum mansoni* is an infrequent parasitic infection caused by the plerocercoid larva of *S. mansoni*. Although in humans sparganosis is usually observed as a subcutaneous nodules somewhere on the body, it is less frequently observed as an internal infection of the eye, brain, or lung (1–3). The plerocercoid lacks reproductive function and is incapable of further proliferation in the intermediate host. Therefore, the only curative treatment is to surgically extract the worm. Here, we present a case of *S. mansoni*, for which smoke-dried boar was the possible source of infection. Furthermore, immunoserological examination by ELISA was shown to be useful for confirmation of a cure.

## Case Report

**Fig. 1:**
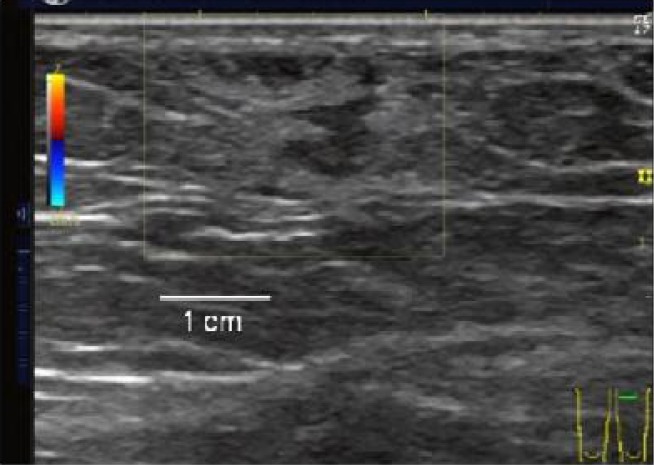
Sparganosis. Ultrasonography shows a serpiginous hypoechoic structure and this compartment is surrounded by a hyperechoic area

In June 2011, a 61-year-old female kindergarten teacher visited our hospital because of a subcutaneous nodule with no inflammation in the left inner aspect of the thigh, which had gradually been increasing in size for a year and a half. Her history revealed that she consumed smoke-dried boar a few years ago. On ultrasonography, a hypoechogenic structure, measuring 2.0cm×2.0cm in size adjacent to a hyperechogenic area was observed (Fig. 1).

**Fig. 2:**
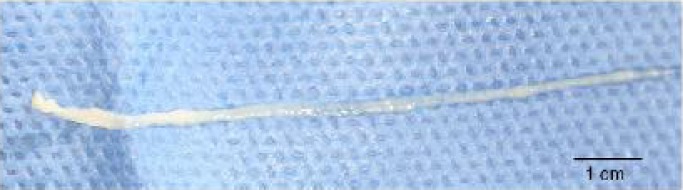
*Sparganum* removed from the lesion in the patient's thigh

**Fig. 3:**
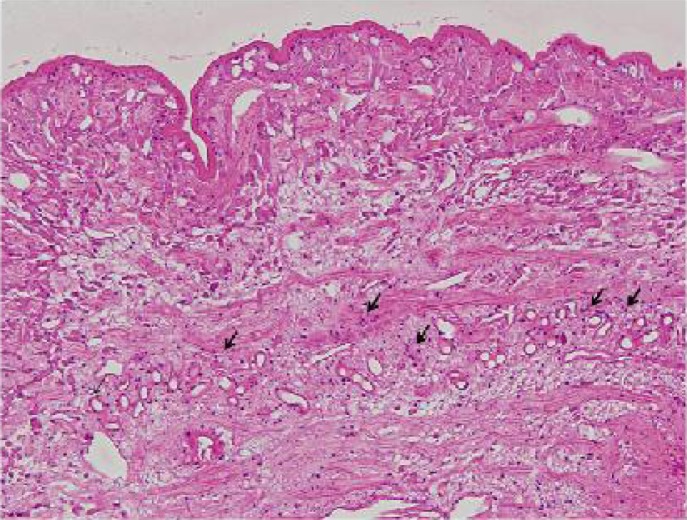
Cross section of sparganum. Many calcareous corpuscles were observed in the cavity (arrows) (H&E × 100)

**Fig. 4:**
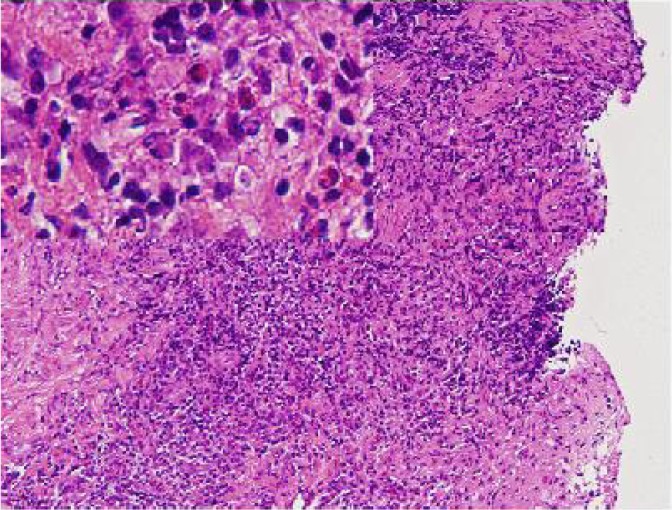
The plerocercoid was surrounded by fibrous tissue and mixed inflammatory cell infiltrates, including lymphocytes and eosinophils. (H&E, ×100 and ×400 [inset])

She had no other symptoms. Hematological examination revealed no abnormal findings, and peripheral eosinophilia was not observed (WBC, 6590/mm^3^; eosinophils, 0.5%). Total surgical excision was performed with the aim of both diagnosis and treatment. A long, slender, whitish, non-vermigrade worm, approximately 10 cm in length and 5 mm in width was removed (Fig. 2). The worm, along with peripheral tissue, was presented for pathological confirmation. Histopathologically, the worm had a non-cellular eosinophilic tegument and subtegumental cells inside the wall. The parenchyma was composed of parechymal cells, calcareous corpuscles, excretory ducts and muscle fibers (Fig. 3). The worm was surrounded by fibrous tissue, lymphocytes, and eosinophils (Fig. 4). Eosinophil infiltration was moderate.

**Fig. 5:**
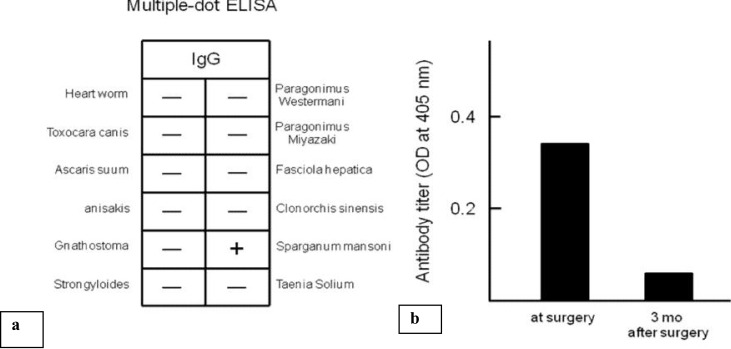
(a) The results of a multiple-dot enzyme-linked immunosorbent assay (ELISA) for the diagnosis of parasitic disease (b) Anti-*sparganum mansoni* IgG antibody titers measured by microplate ELISA. Antibody titers are expressed as the optical density (O.D.) at 405 nm. A value of 0.1 or less was considered nonspecific

These clinical and histological findings were consistent with *S. mansoni*. Soon after parasite infection was suspected, on the same day as the biopsy, her serum was examined using a multiple-dot enzyme linked immunosorbent assay (dot-ELISA) for the diagnosis of parasitic diseases. Because her serum was positive for *S. mansoni* in this assay (Fig. 5a), we tested for parasite-specific IgG antibody using a microplate ELISA for further investigation. The result clearly showed that her parasite-specific IgG antibody titer was high (Fig. 5b). She was concerned about whether another worm was still living in her body after the surgery. Therefore, we measured the IgG antibody after 3 months after the surgery without oral treatment for the parasite. The serum titer of the parasite-specific IgG antibody had decreased significantly (Fig. 5b). We accordingly established that the patient was free of the disease without any further oral treatment.

## Discussion

*Spirometra mansoni* is a relatively rare parasitic infection in human caused by the plerocercoid larva of *S. mansoni*. The adult of *S. mansoni* is naturally parasitic in the intestine of foxes, dogs, cats, raccoons, and tigers. Humans can become infected in 3 different ways: (i) by drinking water containing infected plankton, which are an initial intermediate host; (ii) eating the raw or partly-cooked flesh of a second intermediate host such as snake, bird, deer, horse, boar, and frog; and (iii) putting a peltry from an infected snake or flog on injured skin or a mucous membrane (which is used as a folk medicine in some countries) (4).

In discussing the risk of acquiring an infection in this case, she had no record of overseas travel or an opportunity to directly drink impure water. Because the patient consumed smoke-dried boar a few years ago, it is possible that she became infected by ingesting plerocercoid larvae. According to a study by the National Institute of Infectious Diseases in Japan, 40% of patients with sparganosis were infected due to a diet of raw snakes or frogs (5). Infection from raw boar accounts for less than 2% of all infections (5). Although we could not obtain additional information of the dry smoking method, there is a risk of infection if the worm is not killed by dry smoking at low heat.

Because of their migratory nature, *Sparganum* worms are frequently detected in the subcutaneous tissue of the chest, abdomen, and thigh (6). Such a nodule is clinically suspected as an epidermal cyst, metastatic skin carcinoma, or soft tissue tumor.

In general practice, a definitive diagnosis can be established by histopathology of the worm in the parasitic lesion following surgical removal. In addition, measurement of serum antibody titer by ELISA, which is readily measurable in Japan, is useful as an adjunct to diagnosis or in follow-up assessment. Because the ELISA has high sensitivity and specificity for human sparganosis mansoni (7), when a piece of worm remains in the body after surgical removal or medical treatment, or if 2 or more worms were present, the serum antibody titer does not decrease. In a study of 15 patients with *S. mansoni*, all had high titers of serum antibody (8). In contrast, eosinophilia or increased IgE levels was observed in only 2 patients. A patient becomes negative for this antibody approximately 4 to 6 months after treatment (8). Because serum antibody titer was attenuated 3 months after surgical treatment in our case, the curative approach could be considered successful.

On the basis of our experience with this case, we concluded that quantitative ELISA for sparganosis could be helpful for correct diagnosis or confirmation of cure following a therapeutic course. Therefore, we suggest that the quantitative ELISA should be regularly applied in patients with sparganosis for the evaluation of cure in clinical practice.
